# Real-world performance of the inflammadry test in dry eye diagnosis: an analysis of 1,515 patients

**DOI:** 10.1007/s00417-025-06760-6

**Published:** 2025-03-05

**Authors:** Germán Mejía-Salgado, William Rojas-Carabali, Carlos Cifuentes-González, Laura Zárate-Pinzón, Camilo Andrés Rodríguez-Rodríguez, Guillermo Marroquín-Gómez, Martha Lucía Moreno-Pardo, Juliana Tirado-Ángel, Alejandra de-la-Torre

**Affiliations:** 1 Neuroscience Research Group (NEUROS), Neurovitae Center for Neuroscience, Institute of Translational Medicine (IMT), Escuela de Medicina y Ciencias de la Salud, Universidad del Rosario, Carrera 24 # 63 C 69, Bogotá, Colombia; 2 Health Sciences Faculty, Universidad Autónoma de Bucaramanga UNAB, Bucaramanga, Colombia; 3 Ophthalmology Interest Group, Escuela de Medicina y Ciencias de la Salud, Universidad del Rosario. (OIG UR), Universidad del Rosario, Bogotá, Colombia; 4 Department of Biomedical Informatics, Lee Kong Chian School of Medicine, Nanyang Technological University, Singapore, Singapore; 5 Department of Ophthalmology, National Healthcare Group Eye Institute, Tan Tock Seng Hospital, Singapore, Singapore; 6 HORUS, Grupo Oftalmológico, Ltda, Bogotá, Colombia

**Keywords:** Dry eye disease, InflammaDry test, Matrix Metalloproteinase-9, Ocular surface, Tear film, Diagnosis

## Abstract

**Purpose:**

To assess the diagnostic performance of the InflammaDry test in diagnosing dry eye disease (DED) using different diagnostic criteria and across varying severities.

**Methods:**

A retrospective study was conducted on 1,515 patients. Subjects were categorized into three groups: Group (1) DED based on Dry Eye Workshop-II (DEWS-II): Ocular Surface Disease Index (OSDI) ≥ 13 and at least one abnormal clinical sign (non-invasive tear break-up time [NIBUT] < 10 s, osmolarity > 308 mOsm/L, or corneal/conjunctival staining). Group (2) DED based on criteria used in prior clinical trials: OSDI > 13, Schirmer < 10 mm in 5 min, NIBUT < 10 s, and keratoconjunctival staining. Group (3) Healthy controls: OSDI ≤ 7, NIBUT ≥ 10 s, Schirmer *≥* 10 mm, and no keratoconjunctival staining. DED severity was classified using the ODISSEY European Consensus Group’s definitions into severe and non-severe. Sensitivity, specificity, and predictive values were calculated for both criteria.

**Results:**

1,363 patients were included in Group 1, 401 in Group 2, and 152 in Group 3. Sensitivity was 81.30% in the population diagnosed using previous clinical trial criteria but decreased to 69.99% when applying the DEWS-II criteria. Specificity was 38.16% in both groups, with 409/467 false negatives respectively.

**Conclusion:**

InflammaDry shows good sensitivity in detecting DED in highly symptomatic cases with multiple clinical signs, but its performance decreases when broader criteria like DEWS-II are used. While valuable for detecting inflammation, routine use for DED diagnosis may lead to false negatives, especially in milder cases.

**Supplementary Information:**

The online version contains supplementary material available at 10.1007/s00417-025-06760-6.

## Key messages

*****What is known***:**

• InflammaDry is a diagnostic test that detects elevated levels of MMP-9, a biomarker associated with inflammation in dry eye disease (DED). The test's sensitivity (85%) and specificity (94%) have been reported in clinical trials involving selected patients with symptoms and various signs of DED. However, its performance in real-world settings appears less robust, with studies indicating that only about 40% of patients with DED test positive for InflammaDry. Nonetheless, these findings are based on studies with small sample sizes, leaving its real-world effectiveness unclear.

*****What is new?***:**

• In a sample of 1,515 patients, we observed that while InflammaDry demonstrated high sensitivity—81.30% for non-severe DED and 86.15% for severe DED in populations with DED based on selected criteria like those included in clinical trials, this study demonstrates that InflammaDry's performance declines when broader diagnostic criteria, such as DEWS-II, are used, with sensitivity dropping to 69.99%.

• Current findings highlight that not all patients with DED exhibit significant inflammation, further supporting the need for tailored approaches in diagnosing and managing DED based on inflammation levels.

**Supplementary Information:**

The online version contains supplementary material available at 10.1007/s00417-025-06760-6.

## Introduction

Dry eye disease (DED) is a multifactorial condition affecting the ocular surface, characterized by an unstable tear film due to either reduced tear production or tear instability [1]. Currently, there is no gold standard diagnostic test for DED. Instead, various tests are employed to evaluate different aspects of the tear film, aiding in the diagnosis. These include the tear film break-up time (TBUT), osmolarity, and ocular surface staining [2]. According to the Dry Eye Workshop II (DEWS-II) report, diagnosing DED requires a combination of symptoms and at least one abnormal diagnostic test. Symptoms are typically assessed using scales such as the Ocular Surface Disease Index (OSDI *≥* 13) or the Dry Eye Questionnaire (DEQ *≥* 6). Tests like meibography and tear meniscus height (TMH) measurement are employed to classify further DED into evaporative, aqueous-deficient, or mixed types [2]. 

Advancements in diagnostic methodologies have introduced tools such as the InflammaDry test, which helps reduce clinicians’ reliance on subjective interpretations [3]. This test quantitatively measures Matrix Metalloproteinase-9 (MMP-9) levels in tear samples, a biomarker strongly associated with inflammation. MMP-9 plays a critical role in promoting the release of inflammatory cytokines and mediators from corneal epithelial cells, perpetuating the inflammatory cycle [4–6]. This inflammation can destabilize the tear film, leading to ocular surface epithelial disease [4–6]. Notably, elevated MMP-9 levels have been significantly correlated with symptom severity scores, decreased low-contrast visual acuity, reduced fluorescein TBUT, and increased corneal and conjunctival staining in patients with dysfunctional tear syndrome (defined as OSDI score > 20, with one or more of the following signs: TBUT ≤ 7 s, punctate corneal fluorescein staining, or Schirmer I score < 10 mm) [7], and DED (defined as dry eye symptoms such as stinging, burning, and/or scratchy sensation and at least one of the following: OSDI > 20, TBUT < 5 s, tear meniscometry test without anesthesia < 5 mm/5 seconds, and corneal fluorescein staining results > 1) [8].

A 40 ng/mL threshold could be used to distinguish between healthy individuals and those with DED, as MMP-9 levels in healthy eyes typically range from 3 to 40 ng/mL [7, 9–11]. In a clinical trial conducted by Sambrusky et al. the InflammaDry test demonstrated high sensitivity (85%, 121 of 143 patients) and specificity (94%, 59 out of 63 controls) for detecting DED (defined by all of the following simultaneously: OSDI scores *≥* 13, Schirmer test results < 10 mm in 5 min, TBUT < 10 s, and the presence of keratoconjunctival staining) when comparing patients with healthy controls (defined by all of the following simultaneously: OSDI scores *≤* 7 or less, Schirmer test results *≥* 10 mm in 5 min, TBUT *≥* 10 s or more, and no keratoconjunctival staining) [3]. However, these stringent criteria often exclude patients with mild to moderate DED, leaving the real-world effectiveness of the test underexplored. Accordingly, our study aims to evaluate the performance of the InflammaDry test using different diagnostic criteria and across varying severities, including real-world clinical settings.

## Methods

A retrospective analysis was conducted on patients who visited a specialized dry eye clinic in Bogotá, Colombia, between January 2014 and December 2022. Only patients for whom the InflammaDry test was performed were included in the study. Patients were excluded if there was incomplete data on non-invasive tear break-up time (NIBUT), osmolarity, or ocular surface staining. Additionally, individuals with atopic conditions were excluded from the analysis. Ethical approval for the study was obtained from the Institutional Review Board of Universidad del Rosario (DVO005 2208–CV1662), and the study adhered to the principles outlined in the Declaration of Helsinki.

### Clinical protocol

As part of the standard clinical protocol, a comprehensive medical evaluation focused on identifying systemic diseases. Conditions such as thyroid disease, Sjögren syndrome (SjD), systemic lupus erythematosus (SLE), connective tissue disorders, atopy, rosacea, psychiatric disorders, and diabetes were explicitly recorded. An ophthalmologic history was also taken, covering details of prior corneal refractive surgeries, eyelid surgeries, and contact lens use. Each condition or history element was documented as either present or absent.

Additionally, all patients completed the OSDI. Subsequently, the ocular surface was evaluated using various diagnostic tools, including the Oculus Keratograph 5 M, Topcon DS3 anterior segment camera, and the Tear Lab Osmolarity System. Details of the techniques employed are provided in **Supplementary Material 1.**

For the InflammaDry test (Inoftal, Quidel Corporation, USA), the operator collected tear samples by dabbing the patient’s lower palpebral conjunctiva 6–8 times with the sample collector without using anesthetic or fluorescein to achieve saturation indicated by a pink or glistening fleece. The saturated fleece was inserted into the cassette, and the tip was immersed in a buffering solution for 20 s. Results were determined after 10 min, with one blue and one red line indicating a positive result (MMP-9 ≥ 40 ng/mL) and a single blue line a negative result (MMP-9 < 40 ng/mL) [3].

### Group definitions

The diagnosis of DED was made based DEWS -II on symptoms (OSDI ≥ 13) and, at last one abnormal sign (NIBUT < 10 s, osmolarity *≥* 308 mOsm/L in either eye or an interocular difference > 8 mOsm/L, ocular surface staining with more than five corneal spots, more than nine conjunctival spots, or lid margin involvement greater than two mm in length and 25% in width) [2] (Group 1). When both eyes fulfilled the criteria, the eye with the more severe disease was selected [12]. If both eyes showed equal severity, one eye was randomly chosen. The definition of DED used in the clinical trial by Sambursky et al. was also applied for comparative purposes (Group 2) [3]. Patients were classified as healthy controls if they had simultaneously an OSDI score ≤ 7, a Schirmer test result of at least 10 mm in 5 min, a NIBUT of 10 s or longer, and no evidence of keratoconjunctival staining, following the exact definition used by Sambursky et al. (Group 3) [3]. Groups were matched by age and sex.

The severity of DED eyes for patients diagnosed by Sambursky et al. criteria disease was further categorized using the ODISSEY European Consensus Group criteria, prioritizing two key factors: patient-reported symptoms and corneal fluorescein staining. Severe DED was defined as an OSDI score ≥ 33 and a corneal fluorescein staining score ≥ 3 on the Oxford scheme. In cases where signs and symptoms were not in agreement, additional criteria such as tear hyperosmolarity (> 328 mOsm/L), Schirmer test results < 3 mm, or the presence of filamentary keratitis, were used to confirm severe disease [12].

#### Statistical analysis

The descriptive analysis reported the quantitative variables as mean and standard deviation (SD) and categorical variables as relatives, absolute frequencies, and percentages. The chi-square (χ2) test was used to compare categorical variables, and the t-test was used to compare continuous variables between the two comparisons: group 1 vs. group 3 and group 2 vs. group 3. Statistical analysis was conducted using Jamovi V2.3.

Sensitivity was determined by the proportion of true positive results (patients correctly identified with DED) among all actual DED cases. Specificity was calculated as the proportion of true negative results (healthy individuals correctly identified as not having DED) among all real healthy cases. The positive predictive value (PPV) and negative predictive value (NPV) were computed to assess the probability that patients with positive and negative InflammaDry test results truly have or do not have DED, respectively. Although the DEWS-II epidemiology report documented a DED prevalence of 50% [13], PPV and NPV were calculated with a prevalence of 72%, which was the prevalence of DED in our database. Lastly, the overall accuracy of the test was determined by the proportion of all correct test results (both true positives and true negatives) in the total study population by calculating the area under the ROC curve (AUC). All analyses were made using MedCalc statistical software (version 22.019) [14]. A broader explanation of metrics and 95% confidence interval calculations is available online [14].

The statistical analysis was conducted across four comparisons: (i) Group 1 vs. Group 3, (ii) Group 2 vs. Group 3, (iii) patients with non-severe DED based on ODISSEY European Consensus Group criteria from Group 2 vs. Group 3, and (iv) patients with severe DED based on ODISSEY European Consensus Group criteria from group 2 vs. Group 3 [2, 3, 13].

## Results

From the 1,515 eyes (1,515 patients) included in the study, 1,363 were classified as having DED based on DEWS-II criteria (Group 1), and 401 patients met the criteria for DED according to the clinical trial definition (Group 2). In contrast, 152 patients were grouped as healthy controls (Group 3). According to DEWS-II criteria, the mean age of the 1,363 patients with DED was 53.9 ± 13.2 years, with 1,146 (84.1%) being female. For the 401 patients diagnosed based on the clinical trial criteria, the mean age was 53.4 ± 14.7 years, with 85.7% female. There were no differences in age or sex between the DED groups and the healthy control group (*p* > 0.05). All patients were Hispanic.

Patients with DED demonstrated a higher prevalence of conditions associated with DED, such as thyroid disease (25.5% in the group diagnosed by DEWS-II vs. 3.9% in the healthy group, *p* < 0.001). SjD was significantly more prevalent in both DED groups compared to the healthy group (*p* < 0.05). Patients from both DED groups exhibited lower NIBUT, increased corneal staining, and conjunctival staining compared to the healthy group (*p* < 0.05). Meibomian gland loss greater than 50% in the inferior eyelid was more frequently observed in the DEWS-II diagnosed group compared to the healthy group (29.4% vs. 23.7%, *p* = 0.005). Tear meniscometry was lower in the clinical trial criteria DED group than in the healthy group (0.20 vs. 0.23, *p* < 0.001). Osmolarity levels did not differ between the DED groups and the healthy control group (*p* > 0.05) **(**Table 1**).**

**Table 1:** Demographic and clinical characteristics between groups

Demographics	DED based on DEWS II (Group 1) *n* = 1,363	DED based on clinical trial criteria* (Group 2) *n* = 401	Controls (Group 3) *n* = 152	*p*-value^a^	*p*-value^b^
Female, n (%)	1,146 (84.1%)	344 (85.7%)	128 (84.2%)	1.00	0.739
Age, mean ± SD	53.9 ± 13.2	53.4 ± 14.7	55.2 *±* 14.6	0.254	0.198
Medical history					
Thyroid diseases, n (%)	347 (25.5%)	105 (26.2%)	6 (3.9%)	**<** 0.001	0.070
Sjögren syndrome, n (%)	209 (15.3%)	73 (18.2%)	3 (2.0%)	0.001	0.011
Connective tissue diseases, n (%)	116 (8.5%)	44 (11.0%)	6 (3.9%)	0.050	0.026
Rosacea, n (%)	77 (5.6%)	22 (5.5%)	2 (1.3%)	0.023	0.118
Refractive surgery, n (%)	274 (20.1%)	88 (22%)	3 (2.0%)	**< **0.001	0.077
Contact lens, n (%)	57 (4.2%)	14 (3.5%)	17 (11.2%)	**<** 0.001	0.297
OSDI scores ± SD	38.4 ± 18.9	38.6 ± 18.9	4.57 ± 1.93	**< **0.001	**< **0.001
Ocular surface signs					
NIBUT **±** SD	9.54 ± 5.21	7.92 ± 3.58	10.72 ± 4.15	0.007	**< **0.001
Osmolarity **±** SD	300 ± 10.7	301 ± 11.2	300 ± 8.63	0.352	0.316
*≥* Moderate corneal staining, n (%)	53 (3.9%)	43 (10.7%)	0 (0%)	**<** 0.001	**< **0.001
> Moderate conjunctival staining, n (%)	175 (1.3%)	91 (22.7%)	0 (0%)	0.022	**< **0.001
> 50% MG loss in the superior eyelid, n (%)	369 (27.1%)	118 (29.4%)	32 (21.1%)	0.099	0.442
> 50% MG loss in the inferior eyelid, n (%)	401 (29.4%)	137 (34.2%)	36 (23.7%)	0.005	0.287
TMH ± SD	0.20 ± 0.08	0.20 ± 0.05	0.23 ± 0.08	0.005	**< **0.001
Schirmer test ± SD	14.8 ± 11.4	11.8 ± 10.3	17.3 ± 4.21	**< **0.001	0.052
Eyes with Sambursky clinical trial criteria and non-severe disease †	-	336 (83.8%)	-	-	-
Eyes with Sambursky clinical trial criteria and severe disease†	-	65 (16.2%)	-	-	-

SD: Standard deviation, DED: Dry eye disease, NIBUT: Non-invasive-tear-break-up time, OSDI: Ocular Surface Index, MG: Meibomian gland, TMH: Tear meniscus height

*: Sambursky clinical trial criteria: OSDI scores of 13 or more, Schirmer test results of less than 10 mm in 5 min, BUT of less than 10 s, and keratoconjunctival staining

†: Disease severity classification followed the ODYSSEY European Consensus Group algorithm recommendations

^a^: Comparison between DED group based on DEWS II vs. healthy group

^b^: Comparison between DED group based on clinical trial criteria vs. healthy group

When analyzing the 401 patients diagnosed with DED using the same clinical criteria as the clinical trial compared to healthy controls, the InflammaDry test’s performance showed a sensitivity of 81.30%. However, specificity was relatively low at 38.16%, with an overall accuracy of 69.22%. Similarly, the PPV was 77.17% (74.74–79.44%), while the NPV dropped to 44.24%. When comparing by severity, the sensitivity of InflammaDry increased from 80.36% in non-severe DED to 86.15% in severe cases **(**Table 2**).**

**Table 2:** Sensitivity, specificity, and positive and negative predictive values

Characteristic	DEWS-II Criteria – Group 1 (1,363 patients with DED vs.152 without DED [Group 3])	Sambursky clinical trial criteria- Group 2 (401 patients with DED vs.152 without DED [Group 3])	Non-severe DED (336 patients with non-severe DED vs. 152 without DED [Group 3])	Severe DED (65 patients with severe DED vs.152 without DED [Group 3])
Positive InflammaDry test	1,048	420	364	150
True Positive InflammaDry test	954	326	270	56
False Positive InflammaDry test	94	94	94	94
Negative InflammaDry test	467	133	124	67
True Negative InflammaDry test	58	58	58	58
False Negative InflammaDry test	409	75	66	9
Sensitivity (95% CI)	69.99% (67.48–72.42%)	81.30% (77.13–84.99%)	80.36% (75.70–84.47%)	86.15% (75.34–94.47%)
Specificity (95% CI)	38.16% (30.41–46.38%)	38.16% (30.41–46.38%)	38.16% (30.41–46.38%)	38.16% (30.41–46.38%)
Positive predictive value (95% CI) *	74.43% (71.88–76.81%)	77.17% (74.74–79.44%)	76.97% (74.47–79.28%)	78.18% (75.35–80.76%)
Negative predictive value (95% CI) *	33.09% (28.45–38.08%)	44.24% (37.31–51.40%)	43.03% (35.97–50.39%)	51.73% (36.12–67.01%)
Accuracy	61.08% (58.57–63.54%)	69.22% (65.18–73.04%)	68.54% (64.22–72.64%)	72.71% (66.27–78.52%)

The ‘positive InflammaDry test’ denotes all samples testing positive for DED, while ‘True Positive’ and ‘False Positive’ refer to correctly and incorrectly identified DED cases. The ‘negative InflammaDry test’ indicates samples testing negative for DED, with ‘True Negative’ and ‘False Negative’ distinguishing between correctly identified healthy cases and missed DED cases. ‘Sensitivity’ measures the test’s ability to identify actual DED cases correctly, ‘Specificity’ evaluates its accuracy in identifying non-DED cases, ‘Positive predictive value (PPV)’ and ‘Negative predictive value (NPV)’ indicate the likelihood that positive or negative test results are accurate, and ‘Accuracy’ reflects the overall correct diagnostic rate of the test

*PPV and NPV were calculated considering a prevalence of 72%

**Fig. 1:**
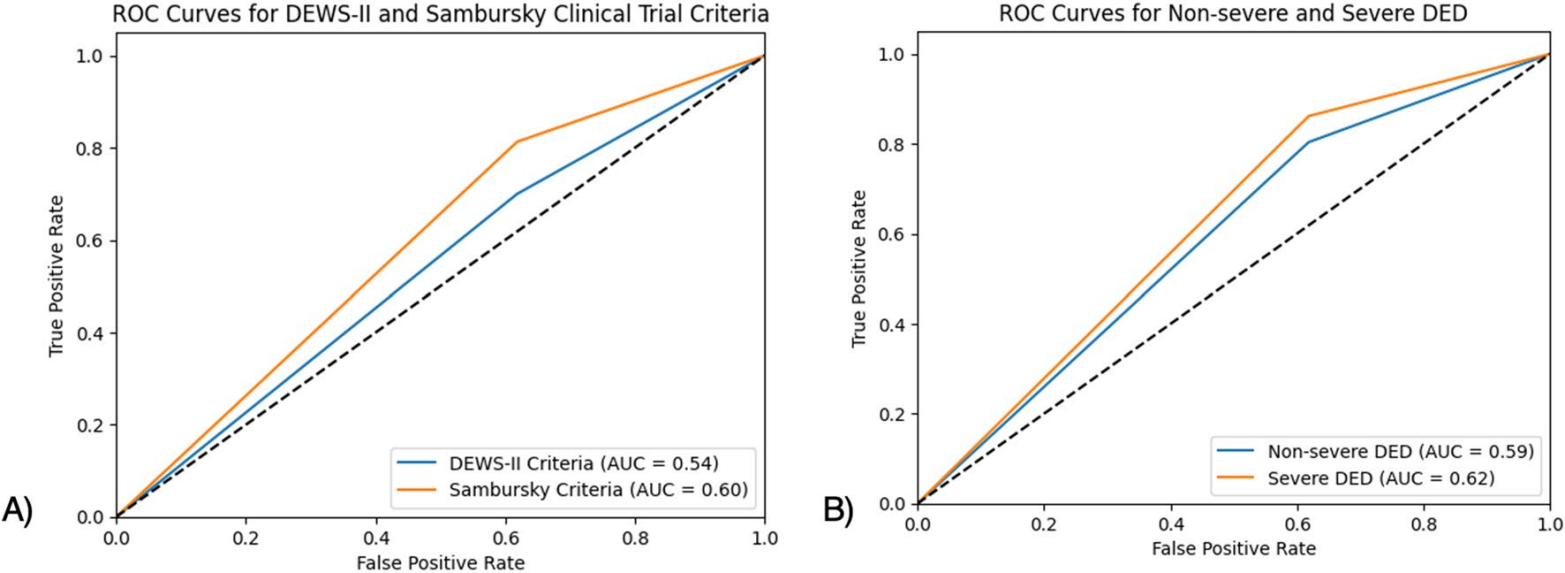
**ROC Curves Comparing InflammaDry Test Performance in Dry Eye Disease according to different diagnostic criteria and across varying severities** **(A)** ROC curves comparing InflammaDry performance for detecting DED using DEWS-II criteria (defined by all of the following simultaneously: OSDI ≥ 13 and at least one abnormal sign: TBUT < 10 s, osmolarity > 308 mOsm/L, or corneal/conjunctival staining) and clinical trial criteria (defined by all of the following simultaneously: OSDI > 13, Schirmer < 10 mm, TBUT < 10 s, and keratoconjunctival staining) compared to healthy controls (defined by all of the following simultaneously: OSDI scores < 7 or less, Schirmer test results > 10 mm in 5 min, TBUT > 10 s or more, and no keratoconjunctival staining). **(B)** ROC curves comparing InflammaDry performance for detecting DED comparing non-severe (OSDI < 33, corneal staining < 3) and severe DED (OSDI ≥ 33, corneal staining ≥ 3) based on ODISSEY European Consensus Group criteria compared to healthy controls (defined by all of the following simultaneously: OSDI scores < 7 or less, Schirmer test results > 10 mm in 5 min, TBUT > 10 s or more, and no keratoconjunctival staining)

Interestingly, when comparing the healthy control group with the 1,363 patients diagnosed using DEWS-II criteria, the overall accuracy decreased to 61.08%, with a corresponding reduction in sensitivity to 69.99%. Additionally, false negatives increased significantly from 75 out of 133 cases to 409 out of 467 cases. The AUC was slightly higher when comparing the population diagnosed with DED based on clinical trial criteria (0.60) versus the DEWS-II criteria (0.54). Furthermore, the AUC was marginally higher in patients with severe DED (0.62) compared to those with non-severe DED (0.59) **(**Fig. 1**).**

## Discussion

Elevated MMP-9 levels have been consistently observed in patients with DED compared to healthy controls across multiple studies, beyond just the clinical trial by Sambursky et al. For example, Chotikavanich et al. measured MMP-9 activity in the tears of 46 newly diagnosed DED patients (defined by an OSDI score > 20 and at least one of the following: TBUT ≤ 7 s, punctate corneal fluorescein staining, or a Schirmer I score < 10 mm) and compared them to 19 asymptomatic controls. Patients with topical medications or other ocular surface diseases were excluded from the study. Their results revealed a significant increase in MMP-9 activity in all patients with DED, regardless of disease severity [7]. Similarly, in a prospective multicenter study involving 237 DED patients, defined by at least one self-reported symptom of dry eye on the OSDI, InflammaDry showed 81% sensitivity and 98% specificity in diagnosing DED [9].

However, not all studies report such high performance for InflammaDry. For example, Messmer et al. document that InflammaDry confirmed DED (defined as three of the following four dry eye criteria: OSDI > 12, TBUT *≤* 10 s, Schirmer test without anesthesia < 10, and corneal staining *≥* 1) in only 40.4% of the patients (19/47) [15]. Similarly, Schargus M et al., in a study conducted involving 20 elderly patients with no prior diagnosis of DED, evaluated them using InflammaDry, the OSDI questionnaire, tear osmolarity, TBUT, corneal fluorescein staining, conjunctival lissamine green staining, Schirmer test, and MMP-9 levels measured via ELISA. DED was defined by two criteria: (1) symptoms alone (OSDI ≥ 10), or (2) the presence of at least two signs such as OSDI ≥ 10, Schirmer ≤ 10 mm, corneal staining ≥ 1, conjunctival staining ≥ 1, and TBUT ≤ 7 s. Among the nine patients identified with DED based on symptoms alone, only one tested positive for MMP-9 via InflammaDry. Among the 14 patients diagnosed with DED based on symptoms and signs, only two tested positive for MMP-9 [16]. Furthermore, Lanza et al., in another study evaluating 128 patients with dry eye symptoms using the DEQ5 questionnaire (DEQ5 ≥ 6), found that only 39% tested positive for MMP-9 with InflammaDry [17]. These results underscore that while MMP levels are elevated in DED compared to healthy populations, not all patients exhibit a significant inflammatory component, and consequently, not all have a positive InflammaDry result.

The current study observed similar sensitivity using the same diagnostic criteria as Sambursky et al. (81.30%) compared to healthy controls. However, the specificity in our study was considerably lower, at 38.16%, with a high rate of false negatives. Several factors could explain this discrepancy. In routine clinical practice, the collection of tear samples may not be as meticulous as in a controlled clinical trial, potentially leading to lower tear volumes being collected, which could result in false negatives despite elevated MMP-9 levels [18, 19]. Additionally, our study included patients who wore contact lenses and those with a history of refractive surgery, groups that were excluded from Sambursky’s trial. These patients were not excluded from our study because our goal was to assess InflammaDry’s performance in a real-world clinical setting, where contact lens use and refractive surgery are common. Indeed, previous studies have shown that patients with post-laser-assisted in situ keratomileusis (LASIK) dry eye display only a 50% positivity rate for MMP-9 with InflammaDry compared to healthy controls [20].

Currently, available commercial tests provide qualitative results (positive or negative) based on a predefined threshold, in this case, 40 ng/mL. A false negative result of InflammaDry should not be interpreted as a test limitation in patients meeting the clinical criteria for DED. Instead, it likely reflects low-grade or absent inflammation on the ocular surface, which does not necessarily rule out the presence of dry eye [15, 17]. In a cross-sectional study by Lee et al. involving 320 patients with DED, a semiquantitative analysis was conducted by subjectively evaluating the intensity of the test line using a four-point scale (0 = negative; 1 = weak positive; 2 = moderate positive; 3 = strong positive). The intensity of the test line showed a positive correlation with corneal staining (*r* = 0.12, *p* = 0.03) and a negative correlation with TBUT (*r*=−0.13, *p* = 0.03). However, no significant correlations were observed when using a quantitative approach with ImageJ version 1.44p to measure intensity objectively [8]. Further studies are needed to assess whether quantitative measurements or establishing thresholds based on clinical severity could enhance the clinical utility of InflammaDry in diagnosis and establishing the severity of DED.

Inflammatory results and MMP-9 activity have been strongly associated with the severity of DED. Chotikavanich et al. identified patients classified in the lowest severity group, based on symptom scores, exhibited significantly higher MMP-9 levels (35.57 ± 17.04 ng/mL, *p* < 0.008) compared to controls (8.39 ± 4.70 ng/mL). In contrast, patients in the highest severity category demonstrated mean MMP-9 activity of 381.24 ± 42.83 ng/mL (*p* < 0.001), significantly higher than controls and all other severity groups [7]. A direct correlation between MMP-9 activity and worsening clinical signs has also been identified. For instance, MMP-9 levels increased as Schirmer test values decreased and osmolarity rose (*p* < 0.05) [21]. Moreover, a study comparing semi-quantitative InflammaDry results found that stronger test reactions (red line’s intensity) correlated positively with higher corneal staining scores (*r* = 0.122, *p* = 0.029) and negatively with BUT (*r*=−0.125, *p* = 0.025) [8]. In the present study, patients with more severe dry eye, whose diagnosis was based primarily on high symptom levels (OSDI ≥ 33) or significant corneal staining (Oxford ≥ 3), showed increased test sensitivity. This aligns with previous literature suggesting that elevated MMP-9 levels contribute to pathological changes in the ocular surface, particularly epithelial damage, destabilizing the tear film and producing irritative symptoms [10, 11, 22, 23].

Notably, when comparing the healthy control group with the DED population based on DEWS-II criteria, sensitivity decreased from 81.30 to 69.99%. This reduction can be attributed to differences in the definitions used. The clinical trial definition followed a more traditional approach, requiring subjects to meet all criteria within strict thresholds, while controls had to fulfill non-overlapping normal criteria. In contrast, DEWS-II requires only the presence of symptoms and one abnormal test result. Although the clinical trial approach yields high sensitivities and specificities compared to DEWS-II criteria, it excludes many DED patients commonly seen in real-world settings. This exclusion introduces sampling bias by preventing the randomization of a broader population [2]. The narrow inclusion criteria can also lead to spectrum bias, as the comparison is made between more severe cases and milder forms of the disease [2, 24]. Both sampling and spectrum bias contribute to the test’s reduced performance in real-world clinical settings.

The study’s strengths include its large sample size, the comparison of varying DED severities, and the use of different diagnostic criteria, including the DEWS-II report, which facilitated the inclusion of a wide range of DED cases. This approach comprehensively assesses the InflammaDry test’s applicability in typical clinical settings. However, some limitations must be acknowledged. The large difference in size between the DED and control groups may lead to bias. However, the main challenge is not the difference in group size but a large number of false negatives, which highlights that not all eyes with DED have an inflammatory component, as previously reported in smaller real-world studies [15, 17]. This finding suggests that InflammaDry may not be suitable as a standalone diagnostic tool for DED. Nonetheless, its ability to identify patients who could benefit from anti-inflammatory therapies remains clinically valuable [4, 20, 25], as these treatments can potentially improve both symptoms and signs in selected cases [26–28].

Additionally, 141 out of 152 (92.7%) patients in the healthy group exhibited Meibomian gland loss, which could potentially introduce bias. However, this criterion was not included in the definition of the healthy group to ensure comparability with the clinical trial by Sambursky et al., the only clinical trial that reports sensitivity and specificity of InflammaDry test, which neither included nor documented Meibomian gland status [3]. Moreover, data on topical medications such as lubricants or anti-inflammatory therapies were not collected. This omission could introduce bias, as these treatments might affect symptoms, signs, and the prevalence of positive InflammaDry results [26–28]. Although this represents a limitation, it also reflects the test’s performance in real-world contexts, where the frequent use of over-the-counter lubricants is a common practice [29].

Furthermore, given the nature and characteristics of the data collected, which were limited to a tear film laboratory within an ophthalmology center, the study is unable to provide precise information on the diagnostic criteria used for patients with SjD (primary or secondary), thyroid disease, and other systemic diseases. Additionally, we are unable to detail the duration of these diseases fully.

In conclusion, these findings suggest that not all patients with symptoms and signs of DED have a positive InflammaDry result. The high sensitivity reported in Sambursky’s study appears to apply primarily to selected cases of DED, particularly those with more than one DED sign, including suggestive signs of elevated inflammation, such as keratoconjunctival staining. However, even within these criteria, the test lacks high specificity, likely due to the wide range of dry eye forms with significant inflammatory components, often associated with systemic conditions such as Meibomian gland disease secondary to rosacea, DED related to SjD, graft-versus-host disease, or post-LASIK dry eye [4, 20, 25]. When less stringent criteria, like those in DEWS-II, are used, sensitivity declines, increasing the likelihood of false negatives in routine clinical practice.

Future research must focus on identifying the clinical phenotypes of DED most strongly associated with a positive InflammaDry test. This would allow clinicians to more accurately target patients likely to benefit from the testing or even from starting anti-inflammatory therapies without MMP-9 testing, ultimately reducing costs. Additionally, this approach could help minimize the risk of false negatives arising from routine testing. Further research should also explore the performance of other inflammatory markers in tears or conjunctiva for DED diagnosis in the real world.

## Supplementary Information

Below is the link to the electronic supplementary material.

ESM 1 (DOCX 18.6 KB)

## Data Availability

The corresponding author can share the information in the databases used in this article upon reasonable request.
