# Supplementary material for: Real-world performance of the inflammadry test in dry eye diagnosis: an analysis of 1,515 patients
Source: Graefes Arch Clin Exp Ophthalmol. 2025 Mar 5;263(6):1623–31. doi: 10.1007/s00417-025-06760-6 (PMC12238174; doi:10.1007/s00417-025-06760-6)
Supplement: Supplementary file 1 — (DOCX 18.6 KB) [file 417_2025_6760_MOESM1_ESM.docx]

**Supplementary material 1: Ocular Surface techniques**

**Determination of NITBUT**

The NITBUT was measured using the Oculus Keratograph 5M. This technique involves projecting a Placido ring onto the ocular surface and focusing it clearly. After the subject completes one blink, they are instructed to stop blinking. The Oculus Keratograph 5M automatically determines two measures of NITBUT: NITBUT-first, defined as the time when the first break-up is detected in any surface segment, and NITBUT-average, determined as the mean break-up time for all surface segments in which break-up has occurred (1).

**Determination of Osmolarity**

Tear osmolarity was measured using the Tear Lab Osmolarity System. The tear collection procedure followed the user manual instructions (2).The patient was seated with their head tilted upwards and eyes looking up. The tip of the osmometer was positioned just above the lower lid in the lateral canthal area until it touched the thin line of the tear film. A beeping sound and the disappearance of the green light confirmed successful tear collection. The osmometer was then placed into the reader within 5–10 seconds, the test card code was selected, the OK button was pressed, and the test result was obtained (2).

**Determination of ocular surface staining.**

Both fluorescein and lissamine green staining were evaluated manually by comparing the pictures of the patients taken with a Topcon DC-3 camera and Oculus Keratograph 5M with the pictures of the Oxford classification. For fluorescein staining, a picture was taken two minutes after instilling a single drop of 0.25% fluorescein sodium in each eye, with a cobalt blue filter in the slit lamp. For lissamine green staining, a single drop of 1% lissamine green solution was applied to each eye, and the subjects were instructed to blink gently. One minute after instillation, a picture was taken and analyzed using a red-free filter in the slit lamp (1).

**Determination of Meibography**

Meibography was performed using the Oculus Keratograph 5M, based on the criteria proposed by Pult and Riede-Pult (3). The evaluation defined the area of Meibomian gland loss (MGL) as the percentage of the area without visible glands in relation to the total visible tarsal area, assigning a score from 0 to 4. Both the upper and lower eyelid Meibomian glands were visualized and graded. A score of 0 represented no atrophy; a score of 1 indicated 0% to 24% MGl; a score of 2 indicated 25% to 49% MGL; a score of 3 indicated 50% to 74% MGL; and a score of 4 indicated > 75% MGL (4).

**InflammaDry Testing**

For the InflammaDry test (Inoftal, Quidel Corporation, USA), the operator collected tear samples by dabbing the patient's lower palpebral conjunctiva 6–8 times with the sample collector without using anesthetic or fluorescein to achieve saturation indicated by a pink or glistening fleece. The saturated fleece was inserted into the cassette, and the tip was immersed in a buffering solution for 20 seconds. Results were determined after 10 minutes, with one blue and one red line indicating a positive result (MMP-9 ≥40 ng/mL) and a single blue line a negative result (MMP-9 <40 ng/mL). InflammaDry positivity was determined at the clinician's discretion.

**Schirmer test**

The Schirmer I test with anesthesia was performed after the InflammaDry test by applying a drop of proparacaine hydrochloride 0.5% in each eye and placing a Schirmer strip in the inferior conjunctival fornix. The measured length of wetting after a 5-minute period was recorded (5).

**References**

1. Kunert K, Sickenberger W. Dry Eye Guide: A Guide to Comprehensive Dry Eye Screening with the OCULUS Keratograph 5M [Internet]. 2017. Disponible en: https://d3ciwvs59ifrt8.cloudfront.net/7c82a155-a2d2-4de8-a159-f406905e28ca/fa6699ea-8ed7-4c98-ac39-b39186a7c94e.pdf

2. TearLab. TearLab Osmolarity System User Manual [Internet]. 2012. Disponible en: https://trukera.com/wp-content/uploads/2022/08/TearLab_Osmolarity_System_User_Manual_-_English.pdf

3. Pult H, Riede-Pult B. Comparison of subjective grading and objective assessment in meibography. Contact Lens and Anterior Eye. 2013;36(1):22-7.

4. Robin M, Liang H, Rabut G, Augstburger E, Baudouin C, Labbé A. The Role of Meibography in the Diagnosis of Meibomian Gland Dysfunction in Ocular Surface Diseases. Trans Vis Sci Tech. 2019;8(6):6.

5. Senchyna M, Wax MB. Quantitative assessment of tear production: A review of methods and utility in dry eye drug discovery. j ocul biol dis inform. marzo de 2008;1(1):1-6.
